# ‘Sheldon' in the Medical Field: Emotional Intelligence and Its Associated Factors in Medical Students of Pakistan

**DOI:** 10.1192/bjo.2022.171

**Published:** 2022-06-20

**Authors:** Unsa Athar, Muhammad Bilal Rehman, Ahsan Nawaz, Aimen Siddique, Ambreen Fatima, Alamgir Aslam Khan, Muhammad Luqman Nagi

**Affiliations:** Shalamar Medical and Dental College, Lahore, Pakistan

## Abstract

**Aims:**

The dearth of emotional intelligence leads to medical students’ inability to handle the pressures of medical education, sub-sequently causing burnout and mental illnesses. Poor emotional intelligence in young doctors also begets increased mistrust from the public, lowering the quality of healthcare delivery. Emotional intelligence of Pakistani students, similar to global context, is impacted by a myriad of psycho-socio-economic factors. It is pertinent to find out the detrimental and/or protective factors, and design interventions to enhance emotional intelligence as a soft skill. With this aim in mind, we explored the relationship of emotional intelligence with adverse childhood experiences and prevalent mental illnesses (depression and anxiety) amongst the medical students of one of the most populous cities of Pakistan; Lahore.

**Methods:**

A cross-sectional study was conducted including currently enrolled MBBS (Bachelor of Medicine and Surgery) students from first year to final year in 2 medical schools of Lahore. An online google form was constructed by combining Modified Adverse Childhood Experiences Score Scale (ACES), Brief Emotional Intelligence Scale (BEIS-10) and Hospital Depression and Anxiety Scale (HADS). Data were exported to SPSS version 25.0 for descriptive and analytical analysis. Pearson's chi-square analysis and logistic regression analysis were used to study the association between the outcome and dependent variables; Odd's ratio (OR) with 95% Confidence Intervals (CI) were calculated.

**Results:**

Participants (N = 324) belonging to two different medical colleges in Lahore, namely King Edward Medical College (public) and Shalamar Medical and Dental College (private), took part in the study.

Pearson's chi-square showed significant association of emotional intelligence with early private schooling (p = 0.029), nuclear family system (0.044) and the presence of symptoms of depression (0.005). The adjusted logistic regression model showed that people who studied in a private sector school (OR: 2.12, CI: 1.01–4.45) and people who lived in a nuclear family (OR: 2.02, CI: 1.00–4.08) had significantly twice the likelihood of having high emotional intelligence. Also noteworthy is that respondents who were depressed according to HADS showed significantly lower emotional intelligence (OR: 0.37, CI: 0.16–0.86)

**Conclusion:**

Emotional intelligence is now being recognized as an important life skill for healthcare providers. Emotional intelligence of medical undergraduates is influenced by a number of factors such as early schooling, family's living situation, current mental health and adverse childhood experiences. More prospective researches should be conducted to evaluate these relationships. Carefully crafted interventions for improving emotional intelligence for medical students must be implied at an early level to achieve better outcomes from medical education.

